# Chitosan-Electrospun Fibers Encapsulating Norfloxacin: The Impact on the Biochemical, Oxidative and Immunological Profile in a Rats Burn Model

**DOI:** 10.3390/ijms252312709

**Published:** 2024-11-26

**Authors:** Corneliu-George Coman, Alexandru Anisiei, Sandu Cibotaru, Daniela Ailincai, Sorin Aurelian Pasca, Caroline Chabot, Ioannis Gardikiotis, Liliana Mititelu-Tartau

**Affiliations:** 1 Pharmacology, Clinical Pharmacology and Algesiology Department, Faculty of Medicine, University of Medicine and Pharmacy “Grigore T. Popa“ of Iasi, 700115 Iasi, Romania; dr.cgcoman@gmail.com (C.-G.C.); lylytartau@yahoo.com (L.M.-T.); 2 Faculté de Médecine, Pharmacie et Sciences Biomédicales, Université de Mons, 7000 Mons, Belgium; 3 “Polycondensation and Thermostable Polymers” Department, “Petru Poni” Institute of Macromolecular Chemistry of Romanian Academy, 700487 Iasi, Romania; 4 Pathology Department, University of Agricultural Sciences and Veterinary Medicine ‘Ion Ionescu de la Brad’, 700490 Iasi, Romania; 5 Department de Radiologie, Cliniques Universitaires Saint-Luc, Université Catholique de Louvain, 1200 Bruxelles, Belgium; 6 Surgery Department, Advanced Research and Development Center for Experimental Medicine ‘‘Prof. Ostin C. Mungiu’’, University of Medicine and Pharmacy ‘‘Grigore T. Popa’’ of Iasi, 700115 Iasi, Romania

**Keywords:** chitosan, electrospinning, nanofibers, burn model, norfloxacin, inflammation, wistar rats

## Abstract

This study investigates the impact of chitosan-based nanofibers on burn wound healing in a rat model. Two formulations of chitosan nanofibers were prepared through electrospinning. The formulations were then incorporated with different amounts of norfloxacin and underwent surface modifications with 2-formylphenylboronic acid. The burn model was applied to *Wistar* male rats by the contact method, using a heated steel rod attached to a thermocouple. The effectiveness of the nanofibers was tested against a negative control group and a standard commercial dressing (Atrauman Ag) on the described model and evaluated by wound diameter, histological analysis and biochemical profiling of systemic inflammatory markers. The results showed that chitosan-based dressings significantly accelerated burn healing compared to the control treatments. The high-concentration norfloxacin-infused chitosan coated with 2-formylphenylboronic acid’ groups exhibited significant improvements in wound closure and reduced inflammation compared to the other groups; antioxidant enzymes SOD and GPx expression was significantly higher, *p* < 0.05, whereas pro-oxidative markers such as cortisol were lower (*p* < 0.05). Macroscopically, the wound area itself was significantly diminished in the chitosan-treated groups (*p* < 0.05). Furthermore, a histological evaluation indicated enhanced epithelialization and granulation tissue formation within the experiment time frame, while the biochemical panel revealed lower levels of inflammatory cytokines and lower leukocyte counts in the treated groups. These findings highlight the potential of the studied chitosan nanofibers as novel nanosystems for next-generation wound therapies, as well as the clinical utility of the novel chitosan fibers obtained by electrospinning technique.

## 1. Introduction

Chitosan is a biopolymer of natural origin which has long proved its excellent capacity for wound healing [1,2,3,4,5]. This is due to its intrinsic properties, such as its hemostatic effect, antimicrobial activity preventing wound infection, and its ability to promote the growth of granulation tissue, thereby accelerating skin regeneration, along with its biocompatibility, low toxicity, biodegradability and mucoadhesion. The chemical structure of chitosan is rich in amine and hydroxyl functional units that allow groups functionalized with other components to enhance certain biological functions. Furthermore, chitosan can be processed into a large realm of biomaterials suitable for application on wounds, such as thin films, membranes, sponges, hydrogels, foams, nanobeads or nanofibers [6]. Vast research on chitosan-based dressings has shown promising results but also disadvantages, leaving room for future development for clinical applications [1,4,5,7,8,9,10,11,12,13,14,15,16,17,18,19,20,21]. Recent review papers have brought to researchers’ attention the necessity of an in-depth investigation of the effect of chitosan on the wound healing process, an aspect which has rarely been addressed in the past [22].

Among chitosan biomaterials, nanofibers appear as a very promising choice due to their easy manipulation, great conformability, semi-permeability for liquid/gas exchange, excellent swelling ability favoring exudate drainage, and good similarity with the extracellular matrix of skin, which facilitates the stimulation of tissue growth [17,18,23]. A plethora of studies dedicated to the investigation of different chitosan-based nanofibers has confirmed the value of chitosan nanofibers, with some reaching the performance required for marketable products for the control of hemorrhages [24,25,26]. The main barrier which needs to be overcome is the processing of neat chitosan nanofibers, a problematic task due to the difficulty of chitosan electrospinning, which necessitates the use of co-spinning agents, i.e., usually synthetic polymers such as poly (ethylene glycol) and poly (vinyl alcohol) of high molecular weight [4]. In using electrospinning with chitosan to yield defect-free nanofibers, the use of high molecular weight synthetic polymers raises suspicions regarding biodegradability, a negative impact on cells and, ultimately, on tissue regeneration [27,28]. Recent studies have evidenced that by using synthetic polymers as sacrificial matrices, it is possible to obtain neat chitosan nanofibers with excellent mechanical properties, making them more suitable for wound healing [17,18,29,30,31].

The scope of the present study was to investigate the impact of neat chitosan nanofibers and their composites with different fillers on a series of parameters which are indicators for wound healing processes. To this end, a series of chitosan-based nanofibers were used as wound dressings in burn wound models on rats and their impact on relevant parameters for wound healing was statistically analyzed.

## 2. Results

### 2.1. Fiber Characterization

**Figure 1:**
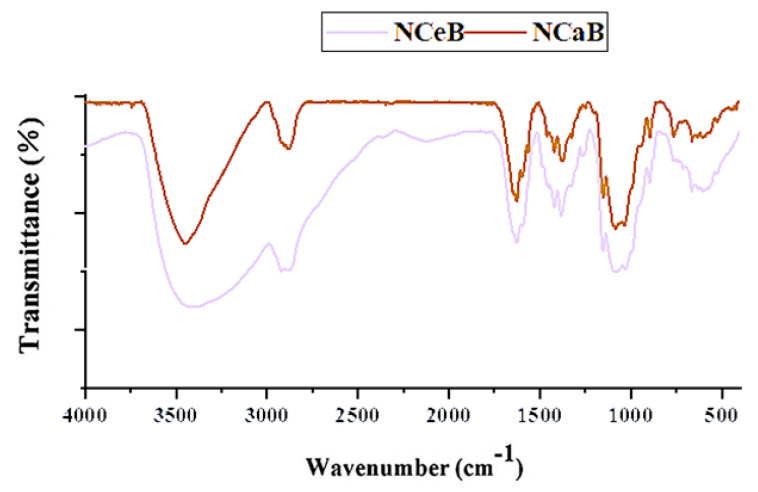
FTIR spectra of the studied fibers.

Neat chitosan nanofibers were prepared by electrospinning chitosan/PEO blend fibers, followed by the selective removal of PEO in water. Then, norfloxacin, known for its broad-spectrum antibiotic properties, was loaded into fibers by immersing in ethanol or water to in order to obtain two different degrees of loading, i.e., 4.35% (‘high concentration norfloxacin’) and 0.94% (‘low concentration norfloxacin’) (Figure 1). Further, the neat chitosan fibers and NFX loaded ones were reacted at the surface with a bioactive aldehyde B via dynamic imine units to reach an imination degree of 10% which proved a good balance between antimicrobial activity and biocompatibility [17,29].

### 2.2. Structural Analysis

**Figure 2:**
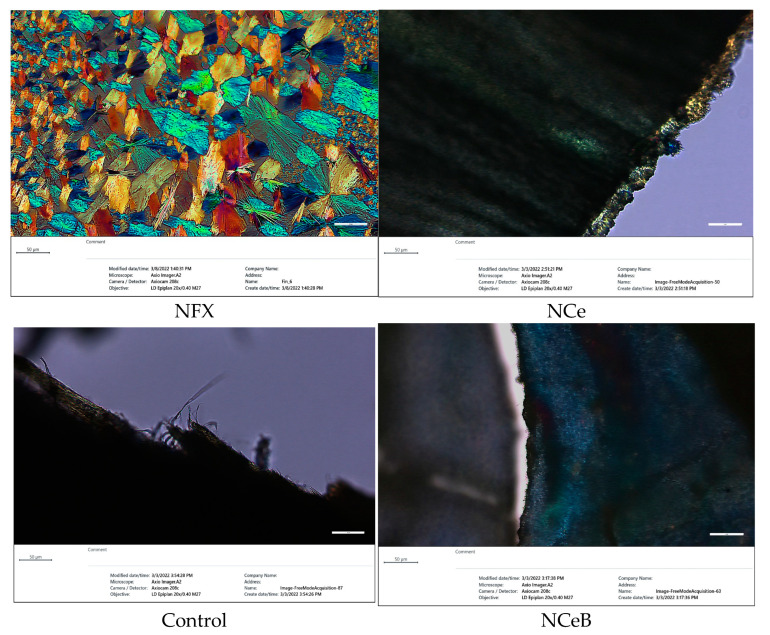
Microphotographs of fibers under polarized light compared with norfloxacin.

The FTIR spectra revealed the presence of norfloxacin and the successful imination of the fibers. Furthermore, POM images of the fibers displayed strong birefringence with iridescent colors, characteristic of norfloxacin (Figure 2). 

**Figure 3:**
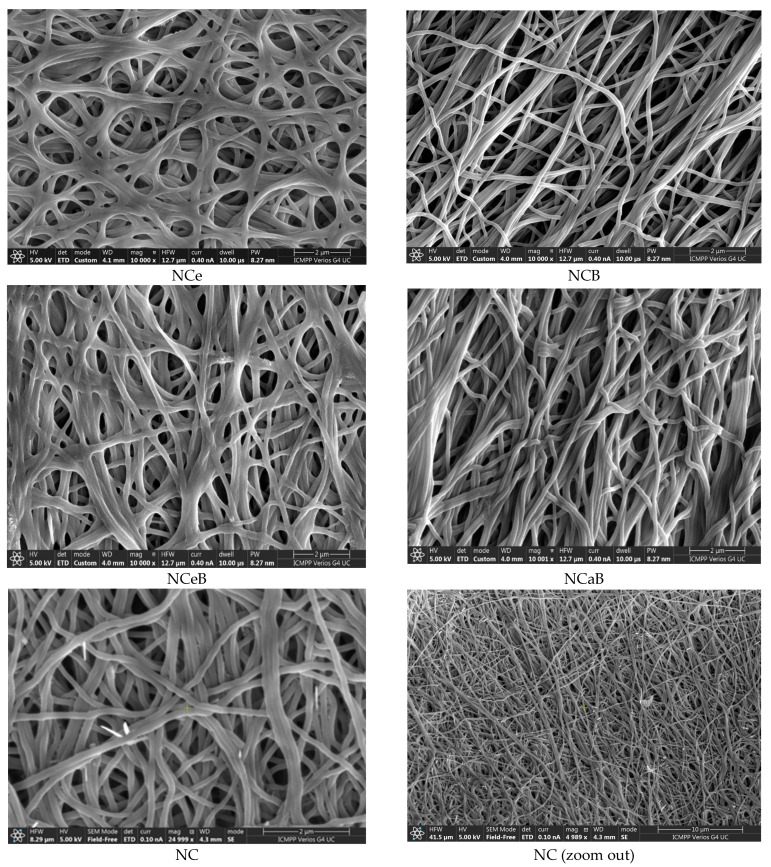
SEM images of the studied fibers.

When NFX is dissolved in ethanol, it undergoes crystallization to form unique and strongly birefringent spherulites, which are characterized by their distinct optical properties. These spherulites exhibit a specific appearance under polarized light, making them easily identifiable. However, in the case of the examined fibers, no such spherulite structures were found on their surfaces. This observation indicates that the drug was not crystallizing on the fibers but was instead likely being encapsulated within the interior of the fiber matrix. This encapsulation process suggests a more intimate interaction between the NFX and the fibers, implying that the fibers served as a carrier for the drug, potentially influencing its release and overall bioavailability. Its ultrastructure can be observed in Figure 3.

### 2.3. Burn Wound Lesion Healing

**Figure 4:**
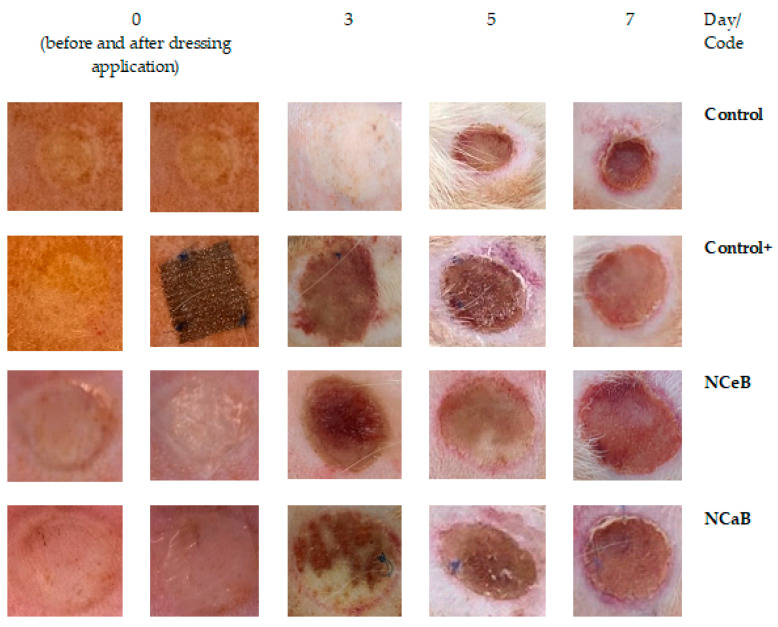
Images of burn wounds collected during the first 7 days after application of the dressing.

Immediately after causing the burns, the wounds appeared round (due to the shape of the steel bar used), slightly deepened and of uniform white color, with an area of moderate erythema and oedema. No blistering was observed, and the boundary between the wound and normal skin was clear. One day after inflicting the burn, in the control group, the occurrence of oedema, marginal erythema and a thin crust in the surrounding area was obvious (Figure 4). This crust became thicker and darker afterwards and developed into an eschar by the 7th day. A substantial increase (* *p* < 0.05) in the area of the burn zone was observed on day 3, followed by contraction of the wound in a time-dependent manner.

### 2.4. Macroscopical Analysis of the Burn Wound

The application of a commercial dressing (Atrauman^®^ Ag, Hartmann, Heidenheim an der Brenz, Germany) led to a slight increase in wound area up to the fifth day, followed by a decrease to its original diameter by the seventh day. Compared to the control group, the reduction in wound size in the positive control burn group was statistically significant (♦ *p* < 0.05), particularly after five days (Table 1). For the tested fibers, the wounds showed no signs of suppuration and appeared rosy, with well-defined edges. By day seven, small shiny areas, indicative of new epithelial cell formation, were visible, suggesting the onset of tissue regeneration, although the lesions had not yet undergone complete healing.

It is important to note that the fibers treated with aldehyde B affected the progression of burn size similarly to the commercial Atrauman dressing, resulting in a statistically significant reduction in wound size (♦ *p* < 0.05) compared to the control group. This effect was particularly pronounced on day 3 of the experiment, with measurements of 1.035 ± 0.01 mm for NCeB and 1.052 ± 0.03 mm for NCaB, as shown in Table 1.

Table 2 depicts the leucocyte changes by subpopulation and day, in all groups. The studied chitosan formulation were statistically significantly lower than the control + group (*p* < 0.05).

Rat serum cortisol was significantly lower in chitosan groups than in the control group, as seen in Table 3 (*p* < 0.05).

SOD enzyme activity is statistically higher in the chitosan groups, as shown in Table 4 (*p* < 0.05).

The impact of the experimental fibers varied based on their composition. The use of chitosan fibers was associated with a rise in cortisol, peaking on day 3 (48.83 ± 2.14 * and 48.83 ± 2.14 *, respectively). Although these values were intermediate between the control and Atrauman groups, they were still statistically significant compared to the control. The slight regulation of cortisol levels by the studied fibers can be attributed to the antioxidant properties of chitosan [32]. In contrast, the nanofibers modified with boronic aldehyde B induced a significant decrease in cortisol levels (34.83 ± 2.04 ♦, 35.33 ± 1.03 ♦, 35.67 ± 1.51 ♦). This was attributed to the strong antioxidant effect induced by the aldehyde B in addition to that of chitosan [33].

In the same trend as SOD, GPx activity is significantly higher in the studied groups than in the control group Table 5 (*p* < 0.05).

Lipid peroxidase is another enzyme whose production is triggered by ROS. This enzyme catalyzes the oxidation of fatty acids in cells, leading to the formation of malondialdehyde (MDA), a substance that can be easily measured in blood and is commonly used as a biomarker for oxidative stress [34,35]. Increased free radicals, typical of acute lesions, result in elevated MDA levels. As expected, the control groups exhibited a statistically significant rise in plasma MDA (*p* < 0.05) compared to baseline, peaking on day 3 (43.5 ± 7.27 vs. 32.33 ± 6.21) (Table 6).

In the positive control group, the MDA increase was significantly lower compared to the control group (34.17 ± 6.58 ♦ vs. 42.83 ± 6.62, ♦ *p* < 0.05), with the peak occurring on day 7, although this increase was not statistically significant compared to the baseline. The fibers’ effects varied based on their composition: fibers without B had lower MDA levels compared to the control but still higher than those in the positive control group. In contrast, fibers containing B showed MDA levels like those of the positive control (Table 6).

The complement serum levels were higher in all treated groups than in the control group, as portrayed in Table 7. 

The capacity of PMN cells to degranulate lytic enzymes that protect against foreign organisms is a critical first line defense feature of the immune system. The recruited macrophages clear wounds of bacterial infections, dead cells and other foreign materials through phagocytosis, which helps control inflammation and initiate tissue remodeling. NBT % serves as an important measure of this capability. In our study, the burns caused a significant increase in NBT % (*p* < 0.05) compared to baseline (14.67 ± 3.42); this was particularly noticeable after 5 days (20.17 ± 4.22) (Table 8).

The use of commercial patches led to a minor and statistically insignificant reduction in NBT % compared to baseline but resulted in a significant decrease compared to the control group (♦ *p* < 0.05), especially on day 5 (15.50 ± 3.82 ♦ vs. 20.17 ± 4.22). The studied fibers provided intermediate reductions in this parameter, with values approaching those of the positive control, particularly with the NCaB fibers after 7 days (Table 8).

### 2.5. Histopathological Evaluation

**Figure 5:**
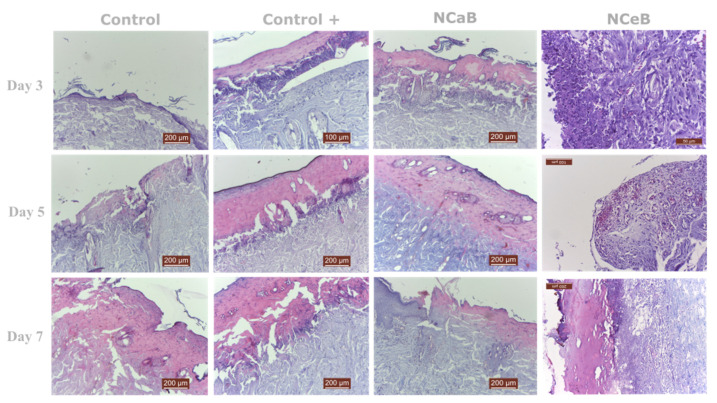
Representative histologic images of burns at 3, 5 and 7 days after the application of the fibers. The sections were prepared at a thickness of 200 µm. The inflammatory cell population is higher in control and control+ groups at all times. NCaB shows a superior cell differentiation at Day 7.

Two rats from each group were euthanized at 3-, 5- and 7-days post-burn induction to assess the microscopic morphology of the wounds. Following euthanasia, the burn sites along with adjacent healthy skin were excised, processed and examined using a digital histological camera. The examination included all skin layers and evaluated burn depth. Local changes in the burn lesion were analyzed, including the presence of inflammatory reactions, vascular damage, necrosis, granulation tissue, connective tissue and re-epithelialization. The findings are detailed in Table 9 and calculated as a modular score in Table 10, with representative images provided in Figure 5. The data indicate that modifying the fiber surface with boronic aldehyde B significantly improved the adverse effects caused by the burn within the first 3 days compared to both the C and C+ model controls. Additionally, NCeB fibers resulted in nearly complete resolution of these effects by day 7.

## 3. Discussions

### 3.1. Fiber Analysis

Modification of the fiber surface with aldehyde B was intended to endow the fibers with antifungal activity [36,37], complementary to the antibacterial activity of drug, as well as to fix the drug on/into fibers and to achieve a prolonged release effect. The success of this strategy was demonstrated by spectroscopic and microscopic methods, which proved the co-existence of the chitosan, NFX and B in the studied fibers (Figure 1).

### 3.2. Structural Analysis

However, spherulites, which are characteristic of norfloxacin crystalized from ethanol, were not observed, pointing to an encapsulation into the fibers, most likely in the pores, resulting from PEO removal. The SEM images also pointed to this conclusion, revealing entangled fibers with diameters of ~170 nm and pores ~4 µm, with no crystals on their surface (Figure 3). Thus, FTIR, POM and SEM images confirmed the presence of norfloxacin in the fibers and their imination with aldehyde B. The FTIR spectra of the studied fibers revealed the encapsulation of NFX by the presence of distinctive bands, i.e., carbonyl stretching vibrations at 1636 cm^−1^ and NH quinolone bending at 1618 cm^−1^ [38], and the reaction of aldehyde B with glucosamine of chitosan by the presence of an absorption band at around 1625 cm^−1^, characteristic of imine bonds, and bands at 1564 cm^−1^ and 760 cm^−1^, which are specific to vibrations of C=C and B-OH groups [29]. All the bands characteristic of chitosan were present in the spectra of composite chitosan fibers, albeit at slightly shifted to lower/higher wavelengths, suggesting physical interactions between the various components (Figure 1) [39].

### 3.3. Biochemical Analysis

#### 3.3.1. Inflammatory Studies

For a deeper understanding of the effect of chitosan-based dressings on the wound healing process, a series of relevant parameters were measured at baseline (day 0) and on days 3–7 after dressing application to assess the impact on the hematological profile, immune system and oxidative stress.

The infliction of the wound resulted in a statistically significant (* *p* < 0.05) increase in the percentage of Ly in peripheral blood compared to the moment before injury (69.2 ± 12.37). This was persistent over 7 days, with a maximum on the third day (79.6 ± 13.25 *) (Table 2). This is in line with the inflammation phase of wound healing [40]. Similarly, an increase of the PMN level with a peak of maximum intensity on the third day (26.6 ± 8.13 vs. 16.0 ± 6.19) (* *p* < 0.05) was noticed. PMNs are considered the first line of defense of the innate immune system, with the role of attacking foreign intruders in the body; these followed the known curve of wound healing. Their local and systemic chemotactic action help recruit other immune cells and trigger the healing processes [41].

Clinical studies revealed that for normal wound healing, a robust PMN response must be limited to the acute wound setting [42]. The application of an Atrauman Ag dressing did not produce significant changes in the level of Ly and PMN compared to the initial moment, having statistically significant changes compared to control M (♦ *p* < 0.05) only on day 3.

A comparison of the samples revealed that the alteration of these two parameters was minimal in the case of the samples containing both NFX and B, indicating a synergistic effect. It can be envisaged that the combined antimicrobial activity of NFX and B improved the anti-pathogen barrier properties of the chitosan nanofibers, with the effect of modulating the PMN activity and consequently reducing their oxidative effect on burnt tissues.

Throughout the experiment, no significant differences were observed in the percentages of eosinophils, monocytes or basophils between the chitosan fiber groups and the controls (Table 2). This indicated that the tested dressings did not induce allergic reactions or other pathological conditions [43,44]. This can be also interpreted as a typical wound healing reaction. Furthermore, consistent with previous research, it can be concluded that the application of these dressings did not impair the normal roles of (i) eosinophils in epithelial remodeling [45,46], (ii) monocytes in regulating wound healing through appetite-related hormones [46] or (iii) the restorative functions of basophils [44].

Cortisol levels are a sensitive indicator of wound healing progress, with elevated cortisol associated with increased oxidative stress and slower healing rates [47]. The analysis of cortisol levels throughout the healing process revealed notable trends (Table 3). As expected, the wound caused a statistically significant rise in cortisol compared to baseline (*p* < 0.01), peaking on day 3 (54.33 ± 1.51 vs. 26.83 ± 2.48). The application of the commercial dressing resulted in only a slight increase in cortisol, with a significant reduction compared to the control group (♦ *p* < 0.05), reaching its peak effect on day 7 (27.83 ± 1.17 ♦).

#### 3.3.2. Oxidative Stress Markers

It is well established that redox signaling and oxidative stress are critical regulators of normal wound healing, supporting processes such as haemostasias, inflammation, angiogenesis, granulation tissue formation, wound closure and extracellular matrix maturation [48]. Reactive oxygen species (ROS) play a vital role throughout these phases, with low concentrations promoting cell survival while inhibiting invasive pathogens. However, injuries lead to increased oxidative stress, which depletes enzymatic antioxidants like SOD and GPx, as they are consumed while neutralizing the elevated ROS levels. Consequently, antioxidant supplementation can prevent cellular oxidative damage and enhance recovery.

SOD, an isoenzyme, scavenges superoxide and increases nitric oxide bioavailability, thereby maintaining vascular homeostasis and contractility [48]. SOD also promotes neovascularization, which aids in the repair of the epidermis and dermis. Dysfunction or reduced levels of SOD can result in severe morphological and cellular defects during wound healing, particularly in older individuals [49]. In acute wounds, SOD activity declines, as it is used to detoxify ROS during the healing process. Thus, SOD levels serve as a key indicator of antioxidant enzyme activity and a measure of the wound healing process.

In the present study, wound formation was accompanied by a significant reduction in SOD levels (*p* < 0.05), falling to almost half of the baseline value (10.67 ± 0.68 vs. 19.33 ± 1.41), with levels progressively decreasing throughout the study (Table 4). The application of the commercial dressing slightly mitigated the decline in SOD, although levels remained significantly lower compared to the initial measurement (* *p* < 0.05) and the control group (♦ *p* < 0.05) at all time points, with the lowest values observed on days 5 (16.33 ± 0.68 ♦ vs. 10.83 ± 1.06) and 7 (16.17 ± 0.71 ♦ vs. 10.67 ± 0.68).

Similarly, the evaluated fibers exhibited nearly identical effects, with those containing boronic aldehyde B showing the closest results to those of the commercial dressing. Nonetheless, the fibers had a less pronounced effect on SOD compared to cortisol, suggesting that while chitosan was more effective at inhibiting ROS, boronic aldehyde B primarily reduced cortisol levels.

GPx is an antioxidant enzyme crucial for maintaining oxidative balance, cellular repair and wound healing across all layers of the epidermis [50]. Since GPx relies on glutathione as an electron donor, reduced glutathione levels in wound lesions inhibit GPx activity. Consequently, monitoring GPx levels is a valuable indicator of wound healing progress. As anticipated, the GPx levels in the control rats decreased significantly over the study period compared to the baseline (*p* < 0.05), reaching their lowest point after 7 days (87.33 ± 27.17 vs. 111.83 ± 20.58).

The use of a commercial dressing notably reduced this decrease (♦ *p* < 0.05), with the lowest value being recorded after 3 days (98.50 ± 25.62). The studied fibers also contributed to reducing the GPx level differences, although the effect varied depending on the composition. Chitosan fibers (especially NCeB) offered only a modest reduction in GPx levels compared to the control, without statistical significance (90.83 ± 26.33 vs. 87.33 ± 27.17 on day 7). However, the presence of B resulted in a significant statistical improvement over the control (101.33 ± 26.62 ♦ vs. 87.33 ± 27.17 on day 7), surpassing the effect of the commercial dressing (Table 5). This underscores the importance of the antioxidant B in supporting effective wound healing.

Normal skin healing involves a complex and delicate interaction between the immune system, keratinocytes and dermal cells [51]. The serum complement system (UCH50) is a crucial component of the immune response [52] and plays a significant role in tissue repair. However, inappropriate activation of this system can lead to impaired wound healing [53,54]. Developing wound dressings that can reduce complement activation is seen as an innovative approach to wound care.

In the control group, a statistically significant decrease in UCH50 was observed compared to baseline, with the most notable drop occurring on day 3 (39.17 ± 8.21 * vs. 53.33 ± 8.62, * *p* < 0.05), indicating inappropriate immune system activity (Table 7). The commercial dressing led to a less pronounced and statistically insignificant reduction. In comparison, fibers without B also reduced UCH50, but their effect was similar to that of the control group. Conversely, fibers containing B achieved UCH50 values comparable to those of the positive group (Table 7).

#### 3.3.3. Limitations

While the preliminary results look promising, there are issues to be addressed in subsequent studies. The short duration of our study was optimal for studying the inflammatory profile, but for long-term effects, we can only extrapolate from the data. A medium term (3 weeks) and long terms (3–6 months) study could have provided a plethora of information regarding the burn scar evolution and its contraction. However, these goals were beyond the scope of the current study. Another limitation was that the animal model, which is a hallmark of burn research, does not translate results directly to the human body. Rats have loose skin with a dermal muscle (‘*panniculus carnosus*’), similar to the facial muscles in human, that increases wound contraction. Furthermore, they have an innate enzyme to synthesize vitamin C, an essential element in collagen synthesis [55,56]. Thirdly, rats’ burns are more resilient to infections than humans. Further study on a different model (pig, monkey) could resemble with more precision a human healing response. However, this pilot study has provided the first stepping-stone for advancing our understanding of the potential of the novel chitosan formulation.

Building on these results, we aim to further our research toward determining the medium-term effects of electrospun chitosan and its encapsulation capabilities. These results would add more clarity to the mechanisms involved in its beneficial effects and could pave the way for superior burn wound dressings.

## 4. Materials and Methods

### 4.1. Substances

Chitosan (126 kDa, DD = 97%) was prepared by alkaline hydrolysis of low molecular weight chitosan (Aldrich, St. Louis, MO, USA). Norfloxacin (98%), poly (ethylene oxide) (1000 kDa), 2-formylphenylboronic acid (97%), acetic acid (99.89%), ethanol (Aldrich, 98.89%) and sodium hydroxide (95%) were purchased from Aldrich and used as received.

### 4.2. Chitosan Dressings Preparation

**Figure 6:**
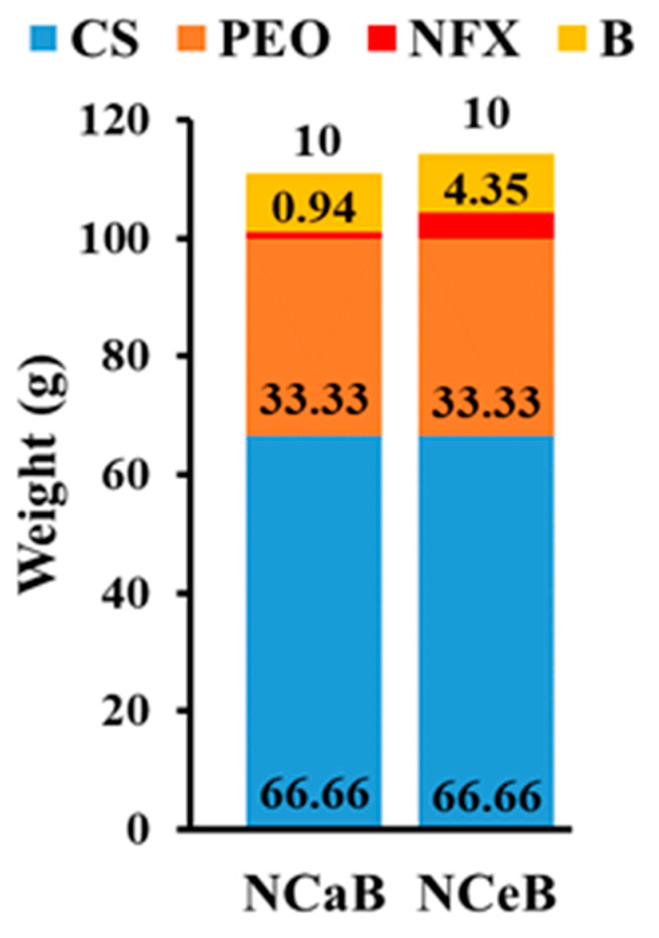
Graphical representation of the fiber composition and the corresponding codes.

Chitosan/poly (ethylene oxide) (CS/PEO) nanofibers were prepared by electrospinning a CS/PEO (2/1, *w/w*) blend solution in 80% acetic acid with an Inovenso NanoSpinner StarterKit, applying the following conditions: 7 kV, 0.4 mL/h; needle-collector distance: 10 cm; inner diameter of needle: 0.8 mm; collector speed: 800 rpm; 27–28°. Next, the PEO was selectively removed by washing in aqueous solutions and the neat chitosan nanofibers were treated to obtain dressings of various compositions, as follows: (1) The fibers were immersed in a saturated solution of norfloxacin (NFX) in ethanol or water for 24 h before being removed and allowed to dry in a normal atmosphere. (2) The fibers encapsulating NFX and neat chitosan nanofibers were sprayed with a solution of 2-formylphenylboronic acid (B) to reach a 10/1 ratio of glucosamine and aldehyde (aldehyde B). The composition of nanofibers is given in Figure 6. It should be highlighted that the content of aldehyde B was determined by UV-vis [17].

### 4.3. Equipment and Analysis

FTIR spectra of the samples were obtained using a VERTEX 70 FT-IR spectrophotometer (Brucker, Karlsruhe, Germany) in ATR mode. The spectra were measured over the range of 4000 to 600 cm⁻^1^, with 32 scans and a resolution of 4 cm⁻^1^. Data processing was performed using OPUS 6.5 software. The fiber morphology was monitored with a field emission scanning electron microscope (SEM) EDAX-Quanta 200 (Thermo Fischer Scientific, Waltham, MA, USA), operated at an acceleration voltage of 20 keV and a polarized optical microscope Axio Imager M2 (Zeiss, Jena, Germany) using cross polarizers. The loading degree of norfloxacin into the fibers was monitored by UV-vis spectroscopy on a Cary 60 UV-Vis (Agilent, Santa Clara, CA, USA) by fitting the maximum absorbance at 272 nm on a previously drawn calibration curve, applying the Lambert-Beer law.

### 4.4. Wound Healing Experiment

**Figure 7:**
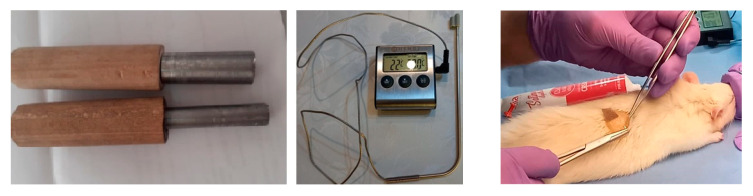
The infliction of burn wound on rats. **Left**: metal contact rods. **Middle**: thermocouple used to confirm the temperature. **Right**: fixing wound dressing on rat burn model skin.

The rats were placed in an aseptic medium, anesthetized with isoflurane. Next, the dorsal part was shaved on a surface of approx. 4 cm^2^ and then depilated to remove any residual hair which may have hindered the occurrence of a uniform burn wound. The skin was disinfected with 70% ethylic alcohol and allowed to dry and reach room temperature for 3 min. Then, a metal rod with a diameter of 1 cm^2^, connected with a thermocouple, was heated at 80 °C in boiling water and then placed on the shaved skin without exerting pressure, supported on its own weight, for 10 s (Figure 7).

After that, the rats were divided into four groups of six rats each, and each group received the nanofibers mats as follows:

- Group 1 (Control): negative reference, bare wound
- Group 2 (Control+): positive reference, covering the wound with a commercial patch (Atrauman Ag^®^, Heidenheim, Germany—polyamide fibers impregnated in triglycerides and silver particles)
- Group 3 (NCeB): wound covered with NCeB nanofibers (high concentration norfloxacin, 4.35%)
- Group 4 (NCaB): wound covered with NCaB nanofibers (low concentration norfloxacin, 0.94%)

**Figure 8:**
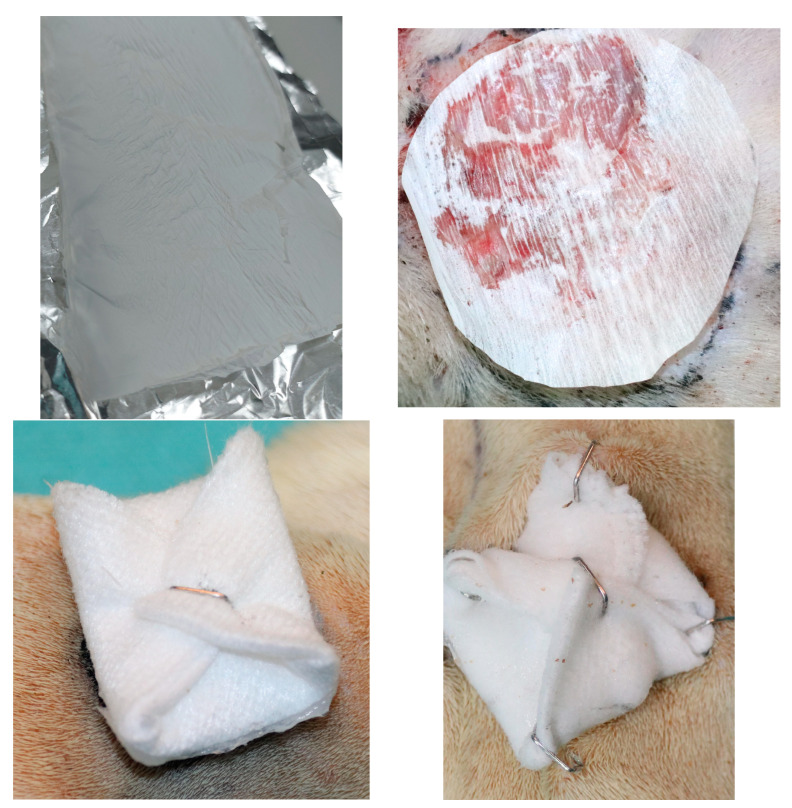
**Upper-left**: Synthesized electrospun chitosan fiber sheet. **Upper-right**: close-up image of chitosan dressing cut, placed in situ before fixation (surgical marker used to trace wound contour for precise area calculation). **Lower left**: cotton gauze folded to the right size. **Lower right**: Same cotton gauze fixed in four points with surgical staples.

The dressing was fixed in place as depicted in Figure 8. The rats were monitored for weight fluctuations and any behavioral signs of discomfort or pain, such as scratching, biting, facial expressions or posture, based on the rat grimace scale [36]. After creating the wound, measurements of its diameter were taken with a ruler on days 0, 3, 5 and 7, alongside observations of local morphological features including general appearance, color, consistency, edges, crust formation and detachment, redness, swelling, discharge, bleeding, granulation and any tissue scarring or infections. Photographs were taken after each assessment for comparative analysis. Visual tracking of wound size was conducted by photographing the wound area from a consistent distance of 35 cm using a digital camera with the same lighting and settings on days 0, 3, 5 and 7. A ruler placed beside the wound provided a reference scale in pixels, allowing wound area calculations through ImageJ software, version 1.8.0.

### 4.5. Blood Markers Examination

Blood samples (0.3–0.5 mL) were collected from the lateral tail vein at two time points: 24 h and 7 days. These samples were analyzed to evaluate the hemodynamic, immune and biochemical profiles. For inflammation studies, the following parameters were measured: number of red blood cells, leukocyte count, cortisol levels, serum complement activity, phagocytic activity of peripheral blood neutrophils (via the NBT test), malondialdehyde (MDA) activity, glutathione peroxidase (GPx) and superoxide dismutase (SOD). Laboratory analyses were performed with the Automated Hematology Analyzer HemaVet 950FS (Drew Scientific, Inc., Boston, MA, USA) and the VITROS 750 XRC analyzer (Alphasoft, Bochum, Germany), using specialized reagents provided by Johnson & Johnson (New Haven, CT, USA).

### 4.6. Histological Examination

At the end of the experiment, the rats were euthanized with 3% isoflurane anesthesia, and tissues from the implant site were collected. These samples were fixed in Bouin’s solution for 24 h and then gradually dehydrated using ethanol baths of increasing concentrations (70%, 75%, 80%, 85%, 90%, 96% and absolute ethanol), with each step lasting three hours [33]. Next, samples were clarified in three successive xylene baths, one hour each, and embedded in paraffin through three paraffin baths at 60 °C.

**Figure 9:**
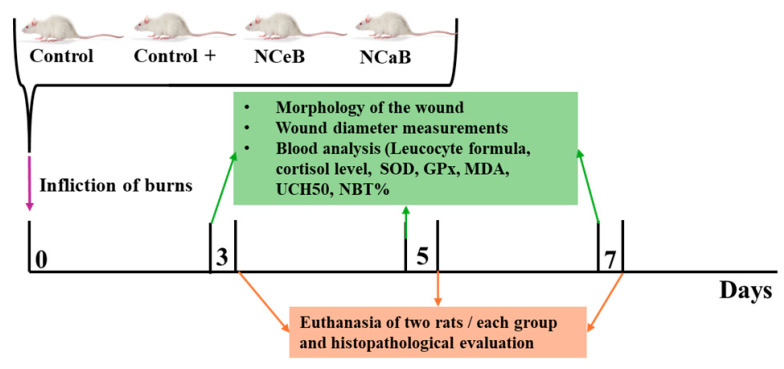
Graphical representation of the in vivo protocol.

The samples were sectioned at a thickness of 5 μm and stained with hematoxylin-eosin (H&E) following standard procedures: xylene deparaffinization, ethanol hydration, hematoxylin staining, eosin counterstaining, alcohol dehydration, xylene clearing and mounting with Canada balsam [38]. Images were captured using a Nikon E600 Eclipse Ti-E Inverted Microscope (Tokyo, Japan), equipped with NIS Elements Basic Research software (https://www.microscope.healthcare.nikon.com/products/software/nis-elements/nis-elements-basic-research, version June 2023) and a Coolpix 950 digital camera (Nikon, Tokyo, Japan). Figure 9 illustrates the in vivo experimental protocol.

### 4.7. Statistical Processing of Data

Data are presented as the arithmetic mean ± standard deviation (S.D.) for each parameter and substance studied. Statistical analysis was performed using SPSS version 17.0 for Windows, employing one-way ANOVA to assess the significance of differences within each animal group and between the test groups and the control. A *p*-value (probability) of less than 0.05 was considered statistically significant.

### 4.8. Ethical Aspects of the Research

The experimental protocol was approved by the Research Ethics Committee of UMF “Grigore T. Popa” in Iaşi (Ethical Approval No. 160/04.03.2022) and authorized by the Veterinary Health Directorate (Project Authorization No. 56/19.02.2022). The research methodology adhered fully to the ARRIVE guidelines and to the European legislation on animal welfare (Directive 2010/63/EU of the European Parliament and of the Council of 22 September 2010, on the protection of animals used for scientific purposes).

## 5. Conclusions

A series of chitosan-based nanofibers were produced by electrospinning using a co-spinning agent as a sacrificial matrix. Norfloxacin antibiotic was embedded into them and their surface was modified with an antioxidant, antifungal aldehyde B. Burn wounds were created on rats with a heated steel rod of 1 cm^2^ diameter. The application of the studied dressings produced a statistically significant diminishing of the wound size. This correlated well with a significant a significant increase in MDA and SOD parameters, suggesting that the antioxidant activity of aldehyde B reduced oxidative stress, favoring wound healing, a result that is consistent with those observed for chitosan-derived compounds.

The statistical insights derived from this study underscore the effectiveness of chitosan-based nanofibers in enhancing wound healing compared to conventional treatments and controls, demonstrating their potential as advanced therapeutic materials for clinical applications in wound management.

## Figures and Tables

**Table 1:** Evolution of wound diameter (cm) during 7 days after dressing application.

Day/Code	Control	Control+	NCeB	NCaB
0	0.968 ± 0.03	0.975 ± 0.01	0.969 ± 0.03	0.976 ± 0.01
3	1.348 ± 0.05 *	1.016 ± 0.03 ♦	1.076 ± 0.01 ♦	1.088 ± 0.01 ♦
5	1.335 ± 0.05 *	0.993 ± 0.01 ♦	1.060 ± 0.03 ♦	1.074 ± 0.01 ♦
7	1.308 ± 0.03 *	0.981 ± 0.01 ♦	1.035 ± 0.01 ♦	1.052 ± 0.03 ♦

* *p* < 0.05 vs. initial moment; ♦ *p* < 0.05 vs. Control.

**Table 2:** Leucocyte count in rats.

Code	Moment of Determination (Day)	Leucocyte Count				
		%				
		PMN	Ly	E	M	B
Control	0	26.6 ± 8.13	69.2 ± 12.37	0.5 ± 0.03	3.5 ± 0.1	0.2 ± 0.03
	3	16.0 ± 6.19 *	79.6 ± 13.25 *	0.4 ± 0.01	3.8 ± 0.05	0.2 ± 0.03
	5	16.8 ± 7.43 *	78.9 ± 11.69 *	0.4 ± 0.01	3.7 ± 0.1	0.2 ± 0.01
	7	18.2 ± 6.38 *	77.3 ± 11.47 *	0.6 ± 0.03	3.7 ± 0.1	0.2 ± 0.01
Control+	0	26.8 ± 6.45	69.0 ± 11.53	0.4 ± 0.03	3.6 ± 0.05	0.2 ± 0.03
	3	26.3 ± 7.37 ♦	69.5 ± 13.19 ♦	0.5 ± 0.03	3.7 ± 0.05	0.2 ± 0.01
	5	26.1 ± 6.29 ♦	69.6 ± 12.55 ♦	0.6 ± 0.01	3.6 ± 0.05	0.2 ± 0.03
	7	26.5 ± 6.11 ♦	68.8 ± 12.43 ♦	0.5 ± 0.03	3.6 ± 0.1	0.2 ± 0.01
NCeB	0	26.8 ± 6.29	68.8 ± 11.35	0.5 ± 0.03	3.7 ± 0.1	0.2 ± 0.03
	3	23.2 ± 6.33	72.5 ± 11.29	0.6 ± 0.01	3.5 ± 0.05	0.2 ± 0.01
	5	23.7 ± 5.51	72.2 ± 11.53	0.4 ± 0.01	3.5 ± 0.05	0.2 ± 0.03
	7	23.7 ± 7.13	72.0 ± 12.41	0.5 ± 0.03	3.6 ± 0.05	0.2 ± 0.01
NCaB	0	26.7 ± 6.67	69.1 ± 10.67	0.5 ± 0.03	3.5 ± 0.1	0.2 ± 0.03
	3	23.0 ± 6.29	72.6 ± 11.55	0.5 ± 0.03	3.7 ± 0.05	0.2 ± 0.01
	5	23.9 ± 6.11	72.0 ± 11.45	0.4 ± 0.03	3.5 ± 0.05	0.2 ± 0.03
	7	24.1 ± 6.39	71.8 ± 11.27	0.4 ± 0.01	3.5 ± 0.05	0.2 ± 0.01

PMN—polymorphonucleates neutrophils, Ly—lymphocytes, E—eosinophils, B—basophils, M—macrophages. * *p* < 0.05 vs. initial moment; ♦ *p* < 0.05 vs. control.

**Table 3:** The influence of norfloxacin nanofibers administration on serum cortisol levels in rats.

Cortisol (µg/dL)				
Moment	C	C+	NCeB	NCaB
0	26.83 ± 2.48	26.67 ± 3.61	26.17 ± 1.60	26.33 ± 2.58
3	54.33 ± 1.51 **	30.50 ± 1.38 ♦	35.67 ± 1.51 ♦	35.33 ± 1.03 ♦
5	53.33 ± 1.86 **	29.17 ± 0.75 ♦	35.17 ± 1.17 ♦	34.67 ± 1.37 ♦
7	52.83 ± 2.14 **	27.83 ± 1.17 ♦	34.83 ± 1.60 ♦	34.33 ± 1.21 ♦

** *p* < 0.01 vs. initial moment; ♦ *p* < 0.05 vs. control group.

**Table 4:** The influence of nanofiber application on SOD activity.

SOD (U/mg Protein)				
Moment	C	C+	NCeB	NCaB
0	19.33 ± 1.41	19.33 ± 1.06	19.33 ± 1.33	19.67 ± 0.95
3	11.17 ± 0.68 *	16.5 ± 1.41 ♦	15.50 ± 1.41 ♦	15.50 ± 0.68 ♦
5	10.83 ± 1.06 *	16.33 ± 0.68 ♦	14.33 ± 0.68 ♦	14.67 ± 0.71 ♦
7	10.67 ± 0.68 *	16.17 ± 0.71 ♦	14.17 ± 0.71 ♦	14.17 ± 0.68 ♦

* *p* < 0.05 vs. initial moment; ♦ *p* < 0.05 vs. control.

**Table 5:** The influence of nanofibers application on the GPx activity.

GPX (mU/mg Protein)				
Moment	C	C+	NCeB	NCaB
0	111.83 ± 20.58	111.67 ± 21.93	112.17 ± 25.58	111.5 ± 25.33
3	87.50 ± 27.53 *	98.50 ± 25.62 ♦	101.67 ± 26.67 ♦	101.50 ± 26.41 ♦
5	87.50 ± 26.82 *	98.17 ± 26.33 ♦	101.83 ± 26.33 ♦	101.50 ± 29.93 ♦
7	87.33 ± 27.17 *	97.67 ± 27.17 ♦	101.50 ± 26.82 ♦	101.33 ± 26.62 ♦

* *p* < 0.05 vs. initial moment; ♦ *p* < 0.05 vs. control.

**Table 6:** The influence of fibers application on the MDA activity.

MDA (nmol/g)				
Moment	C	C+	NCeB	NCaB
0	32.33 ± 6.21	32.17 ± 7.17	32.5 ± 6.95	32.33 ± 6.62
3	43.5 ± 7.27 *	34.67 ± 5.93 ♦	38.50 ± 6.37 ♦	38.17 ± 6.43 ♦
5	43.17 ± 6.33 *	34.50 ± 6.45 ♦	38.17 ± 7.43 ♦	37.17 ± 6.58 ♦
7	42.83 ± 6.62 *	34.17 ± 6.58 ♦	37.33 ± 6.17 ♦	36.83 ± 5.82 ♦

* *p* < 0.05 vs. initial moment; ♦ *p* < 0.05 vs. control.

**Table 7:** The influence of fiber application on serum complement activity.

Complement (UCH50)				
Moment	C	C+	NCeB	NCaB
0	53.33 ± 8.62	53.67 ± 9.06	53.50 ± 8.45	53.33 ± 8.29
3	39.17 ± 8.21 *	52.17 ± 8.58 ♦	50.50 ± 8.27 ♦	50.67 ± 7.33 ♦
5	39.67 ± 7.39 *	52.50 ± 8.33 ♦	50.57 ± 8.39 ♦	50.50 ± 7.67 ♦
7	39.50 ± 8.42 *	52.67 ± 8.45 ♦	50.83 ± 8.95 ♦	51.17 ± 8.58 ♦

* *p* < 0.05 vs. initial moment; ♦ *p* < 0.05 vs. control.

**Table 8:** The influence of nanofiber application on NBT %.

NBT (%)				
Moment	C	C+	NCeB	NCaB
0	14.67 ± 3.42	14.83 ± 3.39	14.83 ± 3.37	15.17 ± 4.17
3	19.33 ± 4.06 *	15.50 ± 3.44 ♦	17.67 ± 4.33	17.50 ± 3.67
5	20.17 ± 4.22 *	15.50 ± 3.82 ♦	17.67 ± 3.42	17.67 ± 3.58
7	18.67 ± 3.58 *	15.33 ± 3.67 ♦	17.83 ± 3.27	16.50 ± 3.45

* *p* < 0.05 vs. initial moment; ♦ *p* < 0.05 vs. control.

**Table 9:** Histopathologic evaluation of the burns.

3 Days				
Evaluation Criteria	Code			
	Control	Control+	NCeB	NCaB
**Intensity of Inflammation**				
Congestion	2	2	2	3
Inflammatory oedema	3	2	2	2
Fibrinous exudation	0	0	0	0
Leukocyte infiltrate (neutrophils, macrophages, lymphocytes, histiocytes)	3	4	4	4
Cleansing of the necrosis zone and resorption of the fibrinous matrix	1	2	2	2
Cell differentiation in the wound (endothelial cells, fibroblasts)	1	1	1	1
Fibrillar neogenesis (collagen fibers) and formation of new blood vessels	0	0	0	0
Re-epithelization	0	0	0	0
**5 days**				
**Intensity of Inflammation**				
Congestion	3	2	2	2
Inflammatory oedema	2	1	2	2
Fibrinous exudation	1	1	1	1
Leukocyte infiltrate (neutrophils, macrophages, lymphocytes, histiocytes)	4	3	3	3
Cleansing of the necrosis zone and resorption of the fibrinous matrix	1	2	1	1
Cell differentiation in the wound (endothelial cells, fibroblasts)	1	3	1	1
Fibrillar neogenesis (collagen fibers) and formation of new blood vessels.	1	1	1	1
Re-epithelization	0	0	0	0
**7 days**				
**Intensity of Inflammation**				
Congestion	1	2	1	1
Inflammatory oedema	0	0	0	0
Fibrinous exudation	0	0	0	0
Leukocyte infiltrate (neutrophils, macrophages, lymphocytes, histiocytes)	2	2	2	3
Cleansing of the necrosis zone and resorption of the fibrinous matrix	0	0	1	1
Cell differentiation in the wound (endothelial cells, fibroblasts)	1	2	2	4
Fibrillar neogenesis (collagen fibers) and formation of new blood vessels.	0	2	1	2
Re-epithelization	0	0	1	1

0—absent 1—very low intensity, 2—low intensity, 3—medium intensity, 4—high intensity Orange: inflammatory phase of healing; Green: proliferative phase; Blue: Remodeling phase.

**Table 10:** Histological analysis revealed significant differences in tissue regeneration parameters across treatment groups.

Group	Epithelialization Score (0–3)	Inflammation Score (0–3)	Granulation Tissue Score (0–3)
Control	1.0 ± 0.2	2.5 ± 0.3	1.0 ± 0.2
Control+	1.5 ± 0.2	2.0 ± 0.2	1.5 ± 0.3
NCeB	2.5 ± 0.1	1.0 ± 0.1	2.5 ± 0.2
NCaB	2.7 ± 0.1	0.8 ± 0.1	2.7 ± 0.1

ANOVA results indicated significant differences (*p* < 0.05) between treatment groups, highlighting the superior performance of NCeB and NCaB in promoting epithelialization and reducing inflammation.

## Data Availability

The original contributions presented in this study are included in the article. Further inquiries can be directed to the corresponding authors.
